# Protective Effect of Dizocilpine (MK-801) On TNBS-Induced Experimental Colitis in Mice

**DOI:** 10.22037/ijpr.2019.1100751

**Published:** 2019

**Authors:** Ehsan Motaghi, Valiollah Hajhashemi, Parvin Mahzouni, Mohsen Minaiyan

**Affiliations:** a *Department of Pharmacology and Isfahan Pharmaceutical Sciences Research Center, School of Pharmacy and Pharmaceutical Sciences, Isfahan University of Medical Sciences, Isfahan, Iran. *; b *Department of Clinical Pathology, School of Medicine, Isfahan University of Medical Sciences, Isfahan, Iran.*

**Keywords:** Dizocilpine, MK-801, Ulcerative colitis, Mice, NMDA

## Abstract

Ulcerative colitis is chronic and recurrent disease of the gastrointestinal tract with uncertain etiology and incomplete treatment options. N-methyl-d-aspartate (NMDA) receptor suppression has shown anti-inflammatory effects *in-vitro* and *in-vivo*. The aim of present study was to evaluate the role of dizocilpine (MK-801), a noncompetitive NMDA receptor antagonist, on TNBS (trinitrobenzene sulfonic acid)-induced murine model of colitis. Dizocilpine (0.1, 1 and 5 mg/kg) was given to mice intraperitoneally from 24 h before induction of colitis and daily thereafter for 4 days. Dexamethasone (1 mg/kg) was used as the reference drug. Colitis was induced by intracolonic administration of TNBS/Ethanol (50/50 v/v, 40mg/kg). Animals were sacrificed 5 days after colitis induction and distal colons were examined macroscopically and microscopically. The colonic tissue level of pro-inflammatory cytokines including interleukin 1β (IL-1β), interleukin 6 (IL-6), and tumor necrosis factor-α (TNF-α) were assessed by ELISA. Myeloperoxidase (MPO) level was also measured in colon. Dizocilpine, particularly with intermediate dose of 1mg/kg significantly improved animal’s weight loss as well as macroscopic and microscopic signs of colitis, reduced colonic levels of IL-1β, IL-6, TNF-α and MPO activity. Hence, dizocilpine has significant protective effects in TNBS-induced colitis and NMDA suppression may be a new and effective therapeutic strategy in ulcerative colitis via decreasing in pro-inflammatory cytokine production.

## Introduction

Ulcerative colitis (UC) represents a chronic recurrent inflammation of large intestine. Although exact etiology of UC is unclear, there are circumstantial evidences to link UC to the immune systems imbalance and genetic and environmental factors (1). UC occurs in the gut of susceptible individuals with genetic innate immune defects. Although, there is no conclusive evidence for involvement of a specific bacteria in this disorder, the interaction between the antigen-presenting cells and some bacteria in the colon leads to an excessive activation of local T lymphocytes and subsequently overproduction of pro-inflammatory cytokines such as tumor necrosis factor-α (TNF-α), interleukin-1β (IL-1β), and interleukin 6 (IL-6) (2). The cytokines activate T and B lymphocytes, neutrophils, eosinophils, and basophils and increase T-lymphocytes viability. T-lymphocytes accumulation via increased viability finally causes colon inflammation (2, 3).

Despite the great deal of attention given to this disease during the past years, its pharmacological treatment is not completely established yet. The high frequency of adverse effects and treatment failure and disease relapse, encourage scientists to search new treatments with better outcomes (4).

The N-methyl-D-aspartate (NMDA) receptors are ionotropic glutamate receptors that could be found mainly in nervous system and are involved in controlling of pain perception, synaptic plasticity, and memory function (5). However, recent studies have identified this receptors in several types of immune competent cells such as thymocytes, lymphocytes, and neutrophils (6). Therefore, it has been assumed that NMDA antagonists have the potential to be used in treatment of different inflammatory disorders. Blockade of the NMDA receptor channel with memantine, for example, delays and attenuates the development of arthritis (7). Moreover administration of ketamine, a potent NMDA antagonist, significantly inhibits the early postoperative IL-6 inflammatory response in human (8).Dizocilpine, a highly selective noncompetitive antagonist on NMDA receptors, is an anticonvulsant drug that shares several pharmacological properties with phencyclidine and ketamine (9). 

Many therapeutic potentials have been demonstrated for dizocilpine such as the treatment of traumatic brain injury (10), and neurodegenerative disorders like Alzheimer′s (11) and Huntington′s disease (12). Moreover, it has been established that dizocilpine may has beneficial effects in inflammatory disorders including allergic lung inflammation (13), osteoarthritis (14), and autoimmune encephalomyelitis (15). 

Although dizocilpine has a low therapeutic index, it may clarify the probable role of NMDA receptors in UC pathology and may represent a future therapeutic intervention for inflammatory bowel disease (IBD). Hence, the current study was accomplished to evaluate the protective effects of dizocilpine on TNBS induced UC in mice. 

## Experimental

*Chemicals*

Dizocilpine, hexadecyl trimethyl-ammonium bromide (HTAB), O-dianisidine dihydrochloride, and TNBS were procured from Sigma-Aldrich (Saint Louis, Missouri, USA). Dexamethasone was also purchased from Raha Pharmaceutical Company (Isfahan, Iran). 

The ELISA (enzyme-linked immunosorbent assay) kits for mouse TNF-α, IL-1β and IL-6 were bought from Boster company (Pleasanton, CA, USA). Diethyl ether oxide and formalin solution (35%) were supplied by Merck (Darmstadt, Germany). L-glutamic acid was purchased from MP biomedical company (Netherland). 

*Animals*

Swiss albino male mice (25 - 30 g) were used in the experiments. The animal room was maintained at 22 ˚C – 23 ˚C and 12:12 h light/dark cycle. The mice were fasted 16-18 hours prior to TNBS instillation. Thereafter, the animals were fed with standard pelleted chow and tap water *ad libitum*. All experiments were performed after receiving approval from the Ethics Committee of the Isfahan University of Medical Sciences, Isfahan, Iran.

*Experimental groups*

The mice were randomly distributed into following groups: Sham (normal mice treated with normal saline) and TNBS-induced colitis groups treated with vehicle (colitis control) or with dizocilpine (0.1, 1 and 5 mg/kg, i.p.), L-glutamic acid (2 g/kg, p.o.) or dexamethasone (1 mg/kg, i.p.). L-glutamic acid was used as a NMDA receptor agonist (16). Colitis was induced in mice by a single intra-rectal instillation of 0.1 mL TNBS/Ethanol (50/50 v/v, 40mg/kg). 

Sham group received intra-colonic normal saline (0.9%) in an equivalent volume. Each group composed of 6 mice. The animals were treated once daily starting 24 h before induction of colitis and continued for 4 days until the animals were sacrificed at the day fifth (17).

*Animal weight assessment*

The animals were weighted each day just before the time of drug administration, during the experiments.

*Colitis induction*

The animals were fasted for 16-18 h before the TNBS enema. Acute colitis was induced by intracolonic instillation of TNBS with a 3 cm length tube under ether anesthesia. The animals were then kept in head down position for approximately 90 seconds to prevent the leakage of TNBS solution. Afterward, food and water were *ad libitum* (18).

**Figure 1:**
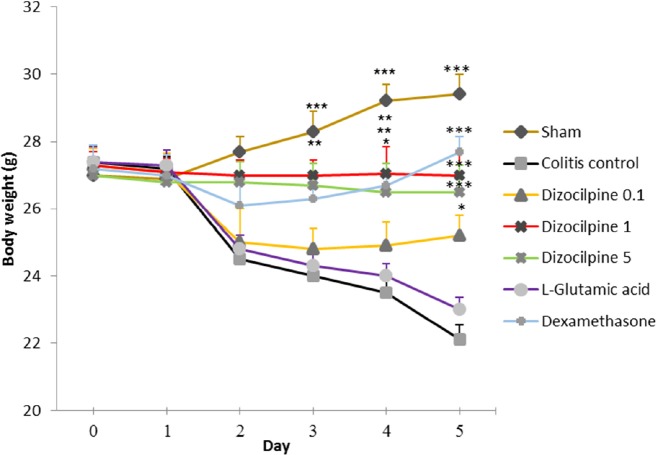
Weight change in mice with colitis treated with dizocilpine (0.1, 1 and 5 mg/kg, i.p.), L-glutamic acid (2 g/kg, p.o.) or dexamethasone (1 mg/kg, i.p.). Animals were treated 24 h prior to induction of colitis and continued daily for 4 days. Data are presented as mean ± SEM. n = 6 per group. ∗ 𝑃 < 0.05, ∗∗ 𝑃 < 0.01, ∗∗∗ 𝑃 < 0.001 *vs *colitis control

**Figure 2:**
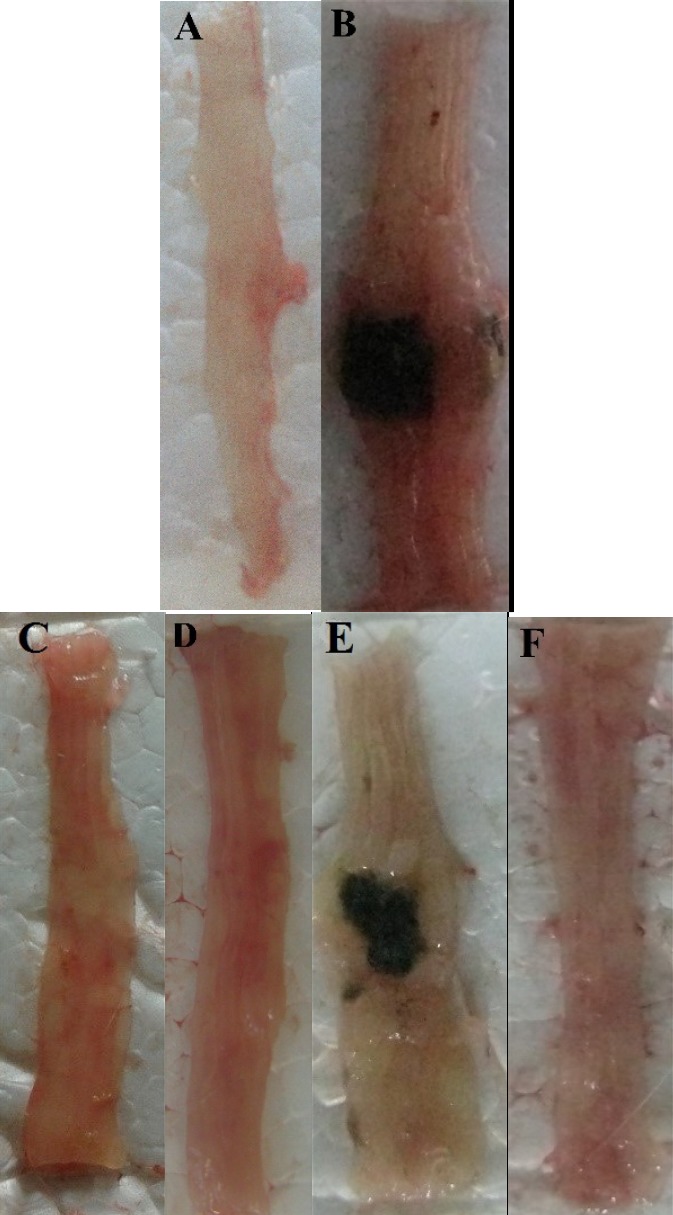
Photos of the distal colon appearance at day 5 after treatment of TNBS-induced colitis in mice**. **(A) Normal colon in Sham group; (B) Colitis control treated with normal saline; (C and D) Colitis treated with dizocilpine Dizocilpine has repeated and one of them should be deleted.(1 and 5 mg/kg, i.p.) (E) L-glutamic acid (2/kg, p.o.) and (F) dexamethasone (1 mg/kg, i.p.).

**Figure 3:**
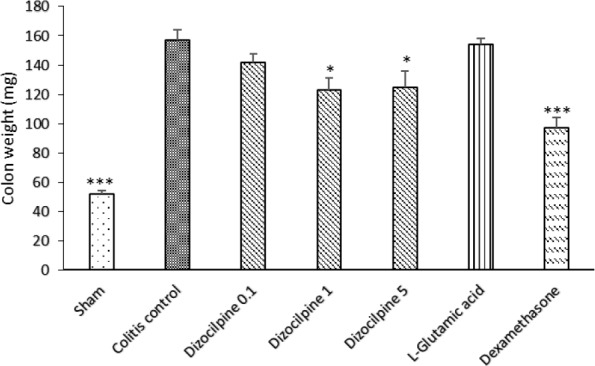
Changes in distal colon weight (3 Cm) of mice with colitis treated with dizocilpine (0.1, 1 and 5 mg/kg, i.p.), L-glutamic acid (2 g/kg, p.o.) or dexamethasone (1 mg/ kg, i.p.). Sham and colitis control groups received an equal volume of normal saline (i.p.). Animals were treated 24 h prior to induction of colitis and continued daily for 4 days. Data are presented as mean ± SEM. n = 6 per group. ∗ 𝑃 < 0.05, ∗∗∗ 𝑃 < 0.001 *vs *colitis control

**Figure 4:**
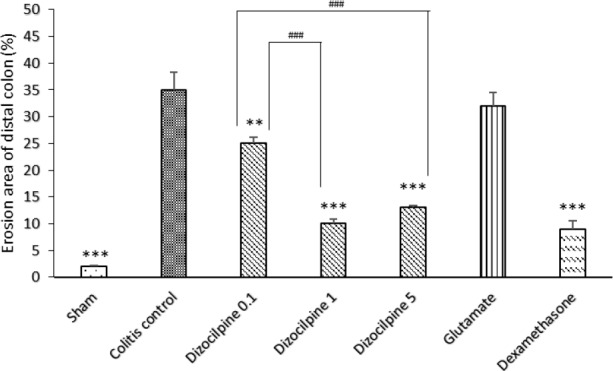
Changes in percentage of erosion area of distal colon (3 Cm) in mice with colitis treated with dizocilpine (0.1, 1 and 5 mg/kg, i.p.), L-glutamic acid (2 g/kg, p.o.) or dexamethasone (1 mg/kg, i.p.). Sham and colitis control groups received an equal volume of normal saline i.p. Animals were treated 24 h prior to induction of colitis and continued daily for 4 days. Data are presented as mean ± SEM. n = 6 per group. **𝑃 < 0.05, ∗∗∗ 𝑃 < 0.001 *vs *colitis control. ### 𝑃 < 0.001compared to dizocilpine 0.1mg/kg

**Figure 5:**
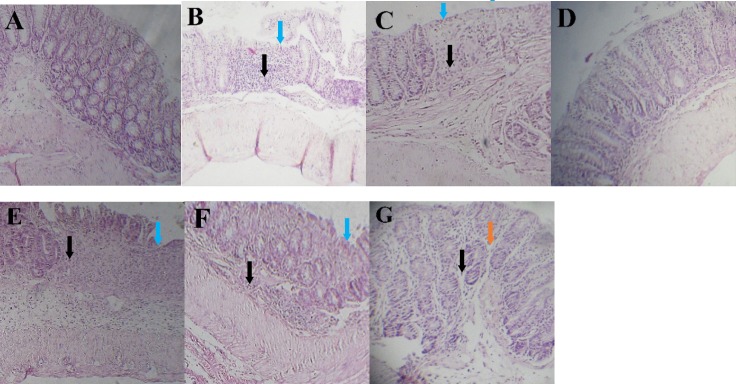
Histopathological appearance of colon sections stained by H&E technique. (A) Sham group, (B and C) Inflamed mucosa in colitis control and L-glutamic acid (2 g/kg) treated groups, respectively. It is associated with mucosal layers destruction, submucosal edema (brown arrows), severely damaged crypt, loss of epithelium (blue arrows) and leukocyte infiltration (black arrows). (d) dexamethasone (1mg/kg, i.p.), (e) dizocilpine 0.1 mg/kg, (f) dizocilpine 1 mg/kg (g) dizocilpine 5 mg/kg (i.p.) improved the extent and severity of the histopathological changes of colitis including inflammation cell infiltration and crypt damages

**Figure 6:**
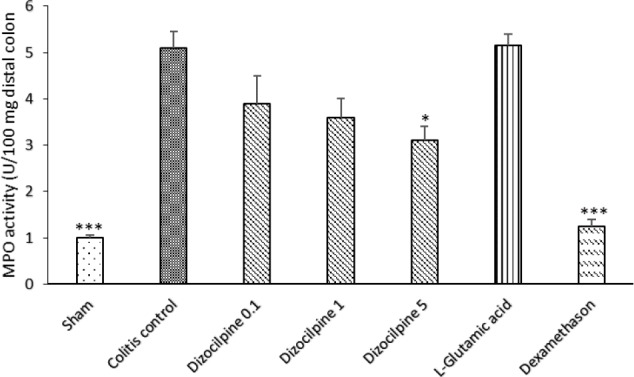
Changes in MPO activity of distal colon of mice with colitis treated with dizocilpine (0.1, 1 and 5 mg/kg, i.p.), L-glutamic acid (2 g/kg, p.o.) or dexamethasone (1 mg/ kg, i.p.). Sham and colitis control groups received an equal volume of normal saline (i.p.). Animals were treated 24 h prior to induction of colitis and continued daily for 4 days. Data are presented as mean ± SEM. n = 6 per group. ∗ 𝑃 < 0.05, ∗∗∗ 𝑃 < 0.001 *vs *colitis control

**Figure 7:**
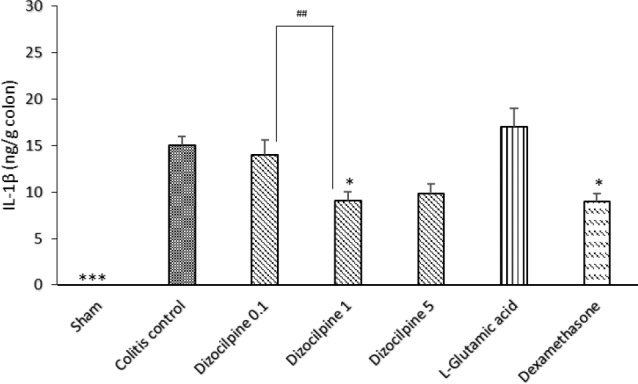
Change in colon IL-1β levels of mice with colitis treated with dizocilpine (0.1, 1 and 5 mg/kg, i.p.), L-glutamic acid (2 g/kg, p.o.) or dexamethasone (1 mg/kg, i.p.). Sham and colitis control groups received an equal volume of normal saline (i.p.). Animals were treated 24 h prior to induction of colitis and continued daily for 4 days. Data are presented as mean ± SEM. n = 6 per group. ∗ 𝑃 < 0.05, ∗∗∗ 𝑃 < 0.001 *vs *colitis control. ## 𝑃 < 0.05 compared to dizocilpine 0.1mg/kg

**Figure 8:**
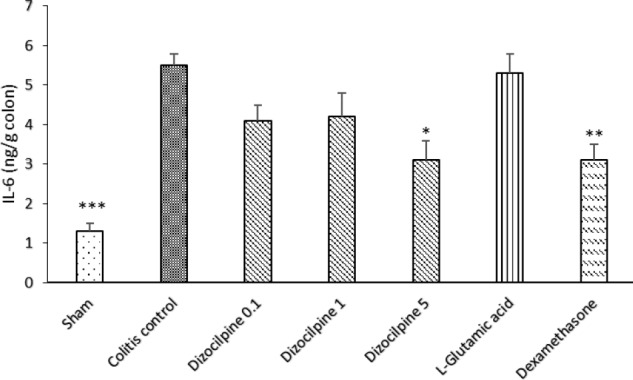
Change in colon IL-6 levels of mice with colitis treated with dizocilpine (0.1, 1 and 5 mg/kg, i.p.), L-glutamic acid (2 g/kg, p.o.) or dexamethasone (1 mg/kg, i.p.). Sham and colitis control groups received an equal volume of normal saline (i.p.). Animals were treated 24 h prior to induction of colitis and continued daily for 4 days. Data are presented as mean ± SEM. n = 6 per group. ∗ 𝑃 < 0.05, ∗∗ 𝑃 < 0.01, ∗∗∗ 𝑃 < 0.001 *vs *colitis control

**Figure 9:**
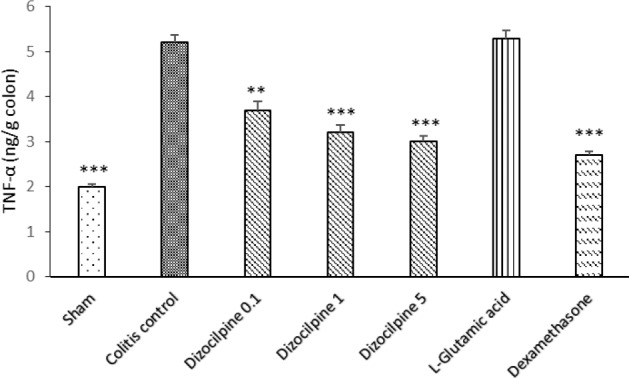
Change in colon TNF-α levels of mice with colitis treated with dizocilpine (0.1, 1 and 5 mg/kg, i.p.), L-glutamic acid (2 g/kg, p.o.) or dexamethasone (1 mg/kg, i.p.). Sham and colitis control groups received an equal volume of normal saline (i.p.). Animals were treated 24 hours prior to induction of colitis and continued daily for 4 days. Data are presented as mean ± SEM. n = 6 per group. ∗∗ 𝑃 < 0.01 and ∗∗∗ 𝑃 < 0.001 vs colitis control

**Table 1:** Effect of dizocilpine (0.1, 1, and 5 mg/kg, i.p.) on macroscopic colonic damage score and pathologic parameters of TNBS- induced colitis in mice

**Groups**	**Macroscopic colitis score (0-10)**	**Microscopic score**			
		**Inflammation** **severity (0–3)**	**Inflammation** **extent (0–3)**	**Crypt damage** **(0–4)**	**Microscopic colitis** **index**
Sham	0 (0-0)^**^	0 (0–0)^**^	0 (0–0)^**^	0 (0-0)^**^	0±0^***^
Colitis control	6 (4-8)	3 (2-3)	3 (2-4)	3 (2-3)	8.33±0.61
Dizocilpine 0.1 mg/kg	5 (4-6)	3 (0-3)	2.5 (1-3)	1.5 (1-2)^**^	5.83± 0.79
Dizocilpine 1 mg/kg	3 (2-4)^**^	2 (1-2)^*^	1.5 (1-3)	1 (0-2)^**^	4.33± 0.76^**^
Dizocilpine 5 mg/kg	2 (0-4)^**^	2 (1-2)^*^	1.5 (1-2)^*^	1 (0-3)^*^	4± 0.45^**^
L-glutamic acid 2 g/kg	5.5 (4-7)	3 (1-4)	2.5 (1-3)	3 (2-4)	8±0.93
Dexamethasone 1mg/kg	4 (2-6)^*^	0.5 (0-1)^**^	0.5 (0-2)^**^	0 (0-1)^*^	1.83±0.75^***^

The values are presented as median (range) for scoring parameters. Microscopic colitis index is presented as mean ± SEM. N = 6 for each group, * 𝑃 < 0.05, ** 𝑃 < 0.01, *** 𝑃 < 0.001 compared to colitis control (n = 6).

*Macroscopic assessment of colon inflammation*

The mice were euthanized by ether, 24 h after final treatment. The distal colon was dissected, opened longitudinally, and then macroscopically scored and its length (cm) and weight (mg) were measured. Macroscopic scoring of colonic ulceration and inflammation was done according to Wallace *et al*. (19). Then, the colon was placed on a nonabsorbent white tissue paper and a digital imaging was recorded by a well-adjusted Canon camera (Powershot G9, 12 megapixel, Japan) to determine erosion area. Fiji-win 32 software was used for analyzing the images to measure erosion area (20). 

Then, the colon sample was cut in tree pieces. One piece was used for colon myeloperoxidase (MPO) activity measurement and other pieces for hematoxylin and eosin (H&E) staining and ELISA evaluation respectively. 

*Measurement of colon MPO activity*

The segment specified for MPO activity assessment was immediately frozen in liquid nitrogen and stored at -70 ºC until measurement of MPO activity according to the method reported by Kim *et al*. (21).

Briefly, the colon sample was weighted and then homogenized in 1 mL of potassium phosphate buffer (pH 6, 50 mm) with 0.5% HTAB on ice by a polytron homogenizer at 30 Hz for 4 min. The homogenate was then centrifuged to obtain supernatant (4 °C, 13400g). Supernatant was collected and frozen at -70 °C until used.

Seven microliters of the sample supernatant fluid were added to phosphate-buffered saline (PBS) containing 0.167 mg/mL O-dianisidine dihydrochloride and 0.0005% hydrogen peroxide. Difference in absorbance at 450 nm was recorded at 30 min intervals for 90 second on a plate reader.

MPO activity was reported as units/100 mg of tissue. One unit of MPO activity was expressed as 1 micromole of peroxide degraded per minute at 25 ^ο^C. 

*Histopathological evaluation of colitis*

Colon samples were fixed in formalin buffered with PBS (10%) and processed routinely and embedded in paraffin. They cut into sections 4 μm-thick using a microtome. The slides were deparaffinized in xylene and counterstained with H&E using standard techniques and scored as described previously (22). 

*Cytokine measurements*

The tissue concentration of pro-inflammatory cytokines including TNF-α, IL-1β and IL-6 were assessed using a commercially obtained ELISA kit (Boster company, Pleasanton, CA, USA) according to manufacturer’s protocols. 

An antibody specific for each murine cytokine was pre-coated onto the microplate supplied in the kits. Briefly, standards, samples and a biotinylated secondary goat antibody were pipetted into the microplate pre-coated with antibodies specific for each cytokine and incubated at 37 °C for 60 min.

After being thrice washed with PBS, plate incubated with the avidin-biotin-peroxidase complex at 37 °C for 30 min. Following 5 times PBS washing, 100 μL of prepared tetramethylbenzidine (TMB) was added to wells. Then, absorbance was read at 450 nm after addition of TMB stop solution.

*Data analysis*

Parametric data have been shown as mean ± SEM and analyzed by one-way ANOVA, followed by post hoc Tukey test. Nonparametric data have been displayed as median (range) and analyzed using Kruskal-Wallis test and subsequently Mann–Whitney U test. A value of *P *< 0.05 was delineated as statistically significant. Statistical analyses were performed with GraphPad Prism (GraphPad software Inc, California, USA). 

## Results

*Effect of dizocilpine on body weight*

Induction of colitis reduced body weight both in colitis control and L-glutamic acid groups. Dizocilpine (0.1, 1 and 5 mg/kg, i.p.) treatment significantly attenuated the weight loss as compared to colitis control. Treatment with dexamethasone reduced body weight loss in mice with colitis too. Nevertheless, L-glutamic acid had no apparent effect on animal’s body weight. Moreover, the animal’s weight increased in Sham group relative to colitis control group (Figure 1).

*Effect of dizocilpine on macroscopic damage of colitis*

TNBS induced sever lesions were associated with edema, hyperemia of the mucosa with large areas of erosion in distal colon while Sham group showed an intact mucosa (Figure 2).

As it is expected, colon damage score (Table 1 and Figure 2), colon weight (Figure 3) and erosion area (Figure 4) increased in colitis control group in comparison with Sham group (*P < *0.001), and dexamethasone as reference drug was effective in reducing the tissue injuries (*P < *0.001). Dizocilpine reduced colon weight (*P < *0.05) and damaging score (*P *< 0.01) at doses of 1 and 5 mg/kg respectively. However, the lowest dose of dizocilpine (0.1 mg/kg) had no significant impact on both parameters. Percentage of erosion area of 3 cm of distal colon decreased in all dizocilpine treatment groups compared with colitis control group. Additionally, the protective effect of dizocilpine at two doses of 1 and 5 mg/kg was significantly more prominent rather than the dose of 0.1mg/kg. L-glutamic acid however, had no significant effect on macroscopic variables of colon inflammation even with the high dose of 2g compared to control (Figures 3, 4).

*Effect of dizocilpine on histopathological features*

The colon mucosa in the Sham group had a normal appearance with intact epithelium and no infiltration of inflammatory cells (Figure 5A). In colitis control group, colon tissues showed loss of goblet cells, submucosal edema, hemorrhage, neutrophils infiltration in the mucosa, inflammation extending to the muscularis mucosae and submucosa, damaged crypts, and destruction of the epithelium (Figure 5B). There was no sign of healing in colonic tissue in L-glutamic acid group compared to colitis control group (Figure 5C). The histologic sections of the dizocilpine (1 and 5 mg/kg) and dexamethasone treatment groups showed ameliorating of histopathologic scores including restoration, progressive re-epithelialization, improvement of the glandular structure, and reduction in edema and congestion in comparison with those of the colitis control group (*P *< 0.05) (Figure 5 D, E, F, G and Table 1). 

*Effect of dizocilpine on MPO activity*

The colitis control group displayed a significant increase in the level of MPO activity as compared with Sham group (*P *< 0.001) (Figure 6). By using dizocilpine, a favorable effect was detectable on MPO activity level in all doses but the magnitude of effect was significant only at 5 mg/kg dose. However, no statistically significant difference was observed between L-glutamic acid group and colitis control group. Moreover, dexamethasone reduced the enzyme activity in significant magnitude (*P *< 0.001). 

*Effects of dizocilpine on the colonic level of pro-inflammatory cytokines*

In contrary to Sham group that showed an undetectable amount of IL-1β by immunoassay, colitis control group expressed a higher colonic level of IL-1β and the anti-inflammatory effects of dizocilpine (1 mg/kg) was confirmed by a significant decrease in IL-1β levels in comparison to the colitis control (*P *< 0.05). Moreover, it was revealed that a significant difference was evident between 0.1 and 1 mg/kg dizocilpine groups (*P *< 0.05) (Figure 7).

In the same manner, IL-6 level was significantly increased in the colitis control group compared to the Sham group (*P *< 0.001). IL-6 levels at doses of 0.1 and 1mg/kg of dizocilpine tend to be declined compared to colitis control group. However, the difference was only significant at the greatest dose (5mg/kg) of dizocilpine (*P* < 0.05) (Figure 8).

Figure 9 is depicting a significant increase in the colonic TNF-α in the colitis control group. Dizocilpine at all doses was effective to reduce the level of TNF- α compared to Sham group.

No significant difference was found for any of the investigated cytokines between L-glutamic acid group and colitis control group. As expected, dexamethasone treatment reduced all investigated cytokines compared to colitis control group (at least *P *< 0.05). 

## Discussion

This study described anti-inflammatory effects of dizocilpine, an uncompetitive NMDA antagonist, on colitis severity. Our study showed that NMDA receptor antagonists could have potential benefit in the management and treatment of UC because our study demonstrated that dizocilpine decreased gross and microscopic parameters of colitis after a four-day treatment.

TNBS induces a chronic colitis resembling human IBD with immunological background and the symptoms of abdominal pain, anorexia, weight loss, anemia, colonic edema, erythema, ulcer, and necrosis (23). Besides overproduction of nitric oxide (24), pro-inflammatory cytokines and MPO (25) occurred in this model.

There are reports suggesting that peripheral NMDA receptors play a key role in peripheral inflammation responses such as T cell development, edema formation, and muscle inflammatory reactions (6, 26). NMDA receptors are frequently detected within the colon (27). So, we suggested that an additional role for NMDA receptors in colitis pathogenesis would be probable. 

Present study showed that dizocilpine, an open NMDA ion channel antagonist, improves colon inflammation parameters including sever weight loss, increased release of pro-inflammatory cytokines and infiltration of inflammatory cells, and distorted mucosal structure in colitic mice. It was found that two graeter tested doses were nearly effective to reduce most colitis markers; however, there was no clear relationship between the dose and related response. By considering the wide range of dizocilpine dosage applied in this study, it is concluded that a ceiling effect could be considered for NMDA implementation in colitis prevention and/or therapy. 

The pathogenesis of UC remains unclear, but overproduction of pro-inflammatory cytokines, such as IL-1β, IL-6, and TNF-α are believed to play a critical role in inflammation formation (28). 

There are several evidences that TNF-α plays a critical role in the pathogenesis of UC especially in TNBS model. TNF- α also induces the release of other cytokines (29). Indeed previous researches on experimental colitis have shown a positive correlation of TNF-α, IL-1β, and IL-6 levels in animal with colitis and the degree of colonic inflammation. So, it is plausible that by inhibition of synthesis or release of these cytokines, dizocilpine could prevent or cause a protection against colitis. 

Results of the present study are in agreement with the previous works in which dizocilpine and other NMDA antagonists such as memantine and ketamine have shown efficacy in preventing inflammatory processes (7, 29). Dizocilpine significantly inhibited IL-1β induced gene expression of cytokines and enzymes involved in cartilage damage and inflammation (14). On the other hand, dizocilpine blocked NMDA receptor-mediated production of nitric oxide and subsequently pro-inflammatory cytokines (30). In another work, memantine attenuated CNS inflammation following endotoxin exposure in rat (31). Ketamine decreased TNF-α release from macrophage in response to bacterial lipopolysaccharides (32). One work also indicated that ketamine decreases *in-vitro* TNF-α secretion into whole blood after exposure to bacterial endotoxins (33). 

Other possible mechanism of anti-colic effects of dizocilpine is via interaction with nitric oxide synthase enzyme by decreasing in calcium entry through NMDA receptor-operated channels (34). This interaction can lead to decrease in level of nitric oxide (NO) related oxidative stress and tissue damage partially mediated by peroxynitrite, a strong and relatively long-lived oxidant that is produced by reaction between NO and superoxide (35). 

NMDA receptors are involved in regulation of intracellular calcium and ROS levels in neutrophils (6). Intraperitoneal administration of dizocilpine decreased neutrophil activation indicated by MPO activity. This result is in accordance with previous reports about effects of NMDA antagonist on neutrophils function (6). 

As noted in introduction, the main etiology of UC is interaction among antigen presenting cells, colon flora, and T lymphocytes. However, how much glutamate and NMDA receptors may be involved in this interaction, remains to be cleared. 

It is worthy to be mentioned that oral administration of L-glutamic acid, a NMDA receptor agonist was not able to elevate or change the colitis markers; likely because of special circumstances such as no requirement for endogenous glutamate for formation of inflammation due to enough activation of NMDA receptors by exogenous glutamate, down regulation of NMDA receptors due to exogenous glutamate administration, limited glutamate delivery to colon due to rapid intestinal absorption and high hepatic clearance. 

## Conclusion

Taken together, dizocilpine showed a protective and anti-inflammatory effect on all features of experimental colitis. It is indicated that NMDA suppression may be a new and effective therapy target in UC patients. However, further investigations are necessary to evaluate whether a similar efficacy can be achieved by other NMDA antagonists and in how much, this mechanism could be effective in clinical setting.
